# Single-session therapeutic plasma exchange as salvage therapy for pegaspargase-induced severe acute pancreatitis accompanied by multiple organ dysfunction in a pediatric patient with B-cell precursor acute lymphoblastic leukemia: a case report

**DOI:** 10.1186/s12887-026-07273-9

**Published:** 2026-08-05

**Authors:** Jianhua Dai, Yingying Xiao, Yueqin Han, Haiyan Ye, Shaohua Xie

**Affiliations:** Department of Pediatrics, Liaocheng People’s Hospital, Liaocheng, Shandong China

**Keywords:** pediatric B-cell precursor acute lymphoblastic leukemia, pegaspargase-induced severe acute pancreatitis, multiple organ dysfunction syndrome, single-session therapeutic plasma exchange, PELOD-2 score

## Abstract

**Background:**

Pegaspargase-related severe acute pancreatitis (SAP) combined with persistent multiple organ dysfunction syndrome (MODS) is a life-threatening adverse reaction in children receiving acute lymphoblastic leukemia (ALL) chemotherapy. Standard intensive supportive care often fails to halt progressive organ damage, while clinical data regarding therapeutic plasma exchange (TPE) as a rescue intervention in pediatric populations remain scarce. Guidelines released by the American Society for Apheresis (ASFA) do not recommend routine TPE administration for all patients diagnosed with acute pancreatitis.

**Methods:**

This retrospective analysis describes a 7-year-old female patient newly diagnosed with B-cell precursor ALL, who developed SAP and refractory MODS 48 h after pegaspargase infusion. SAP diagnosis complied with the 2012 Revised Atlanta Classification, and MODS severity was quantified using the Pediatric Logistic Organ Dysfunction-2 (PELOD-2) scoring system. After 24 h of maximum standardized supportive care failed to reverse clinical deterioration, a single standardized TPE session was implemented. A chronological care timeline and serial laboratory biomarker table were constructed to quantify the dynamic therapeutic response of the patient.

**Results:**

Non-invasive ventilation was applied to relieve intractable respiratory distress; invasive mechanical ventilation and vasopressor support were unnecessary throughout hospitalization. Severe epigastric pain disappeared within six hours after TPE, and respiratory support was successfully withdrawn 24 h post-intervention. Significant declines in pancreatic enzyme levels, extreme hypertriglyceridemia and systemic inflammatory mediators were observed, alongside full recovery of respiratory, renal and hematologic organ function. No adverse events linked to apheresis occurred during treatment. The patient recovered completely from pancreatitis, resumed scheduled maintenance chemotherapy, and maintained minimal residual disease (MRD)-negative hematologic remission at the 3-month outpatient follow-up. Clinical improvement was mainly attributed to extracorporeal clearance of triglycerides and pro-inflammatory cytokines; due to the pharmacokinetic characteristics of pegaspargase at 48 h post-administration, the removal of this chemotherapeutic agent via TPE was negligible.

**Conclusion:**

Single-session TPE can rapidly reverse refractory MODS triggered by pegaspargase-induced SAP when conventional supportive treatments show no efficacy. Nevertheless, complete clinical recovery cannot be solely attributed to apheresis, as continuous multi-organ supportive management was provided throughout the treatment course. TPE can be regarded as a targeted rescue strategy for carefully selected critically ill pediatric patients, yet existing meta-analyses and ASFA consensus statements do not support its universal routine use. Large-scale prospective pediatric cohort studies are required to determine the optimal timing and treatment cycles of TPE.

**Supplementary Information:**

The online version contains supplementary material available at https://doi.org/10.1186/s12887-026-07273-9.

## Introduction

B-cell precursor ALL represents the most common hematological malignancy in children, which differs substantially from solid tumors [1]. Pegaspargase serves as an essential component of modern induction chemotherapy regimens and greatly improves long-term survival rates for pediatric patients. However, chemotherapy-associated acute pancreatitis is an unpredictable and fatal adverse event, occurring in 1% to 3% of children receiving ALL treatment [3, 4]. Based on the 2012 Revised Atlanta Classification, SAP is defined as organ failure lasting more than 48 h; concurrent MODS raises the all-cause mortality rate to over 15% [14]. Chemotherapy-induced gastrointestinal discomfort often delays definitive SAP diagnosis, allowing sustained systemic inflammatory responses to aggravate multi-organ injury [7]. Initial standardized management mainly includes fasting, somatostatin analog infusion, individualized fluid resuscitation and prophylactic antibiotic therapy [13].

TPE interrupts self-amplifying inflammatory cascades by eliminating circulating pro-inflammatory cytokines, lipid-derived toxic metabolites and pancreatic proteases. However, pooled meta-analyses and ASFA guidelines indicate that indiscriminate TPE application cannot reduce overall mortality across heterogeneous pancreatitis patient cohorts [10]. Most pediatric case reports of TPE for asparaginase-related SAP document 3 to 4 consecutive apheresis sessions to stabilize decompensated organ function, and literature describing full MODS remission after only one TPE procedure is extremely limited [8, 13]. This case report adheres to the CARE reporting guidelines, incorporating standardized diagnostic criteria, complete apheresis operation parameters and longitudinal laboratory data to supplement the insufficient pediatric apheresis research focused on asparaginase-mediated pancreatitis.

## Case presentation

A 7-year-old girl weighing 21 kg was newly diagnosed with B-cell precursor ALL and received intravenous pegaspargase at a dose of 2000 IU/m². Forty-eight hours after infusion completion, she developed persistent epigastric pain radiating to the back, recurrent bilious vomiting and sustained fever. The patient had no prior history of chronic pancreatitis, hyperlipidemia, abdominal surgery, drug allergies or hereditary metabolic diseases; no family history of pancreatic disorders or hematological malignancies was recorded.

### Clinical timeline

Day 0: Completion of intravenous pegaspargase infusion.

Day 2: Onset of abdominal discomfort; initial blood tests and contrast-enhanced abdominal computed tomography (CT) were performed.

Day 3: Progressive inflammatory marker elevation, severe hypertriglyceridemia and aggravated dyspnea; admission to the pediatric intensive care unit (PICU), initiation of non-invasive ventilation, PELOD-2 score calculated as 18, consistent with severe MODS.

Day 3 to Day 4: Twenty-four hours of maximal standardized supportive care without clinical or biochemical improvement.

Day 4: Emergency single-session TPE was performed.

Day 5: Successful weaning and discontinuation of non-invasive ventilation.

Day 7: Complete resolution of all MODS manifestations; transfer to the general pediatric hematology ward.

3-month follow-up: Sustained MRD-negative hematologic remission, no recurrent pancreatitis episodes.

### Diagnostic standards and baseline clinical assessments

#### Diagnostic Criteria for SAP (2012 Revised Atlanta Classification [14])

SAP diagnosis requires meeting two of the following three core indicators: characteristic epigastric pain radiating to the back, serum lipase levels three times higher than the institutional upper normal limit, and typical pancreatic inflammatory lesions on contrast-enhanced abdominal CT. Persistent multi-organ dysfunction lasting over 48 h confirmed the diagnosis of SAP. Differential diagnoses including biliary tract obstruction, intestinal obstruction, septic abdominal disease and primary chemotherapy-induced gastrointestinal injury were excluded via imaging and laboratory tests.

#### Diagnostic Criteria for MODS (PELOD-2 Score [15])

The validated PELOD-2 scoring system ranges from 0 to 33, and higher scores correspond to elevated critical care mortality risks. The patient’s score of 18 indicated severe dysfunction limited to the respiratory, renal and hematologic systems; cardiovascular and neurological functions remained stable throughout hospitalization.

#### Manifestations of organ impairment

Respiratory system: Tachypnea, intractable hypoxemia, bilateral moderate pleural effusions and diffuse interstitial pulmonary edema identified on chest CT. Non-invasive ventilation was administered to correct hypoxemia prior to TPE.

Renal system: Oliguria, elevated serum creatinine and transient metabolic acidosis requiring alkaline agent correction.

Hematologic system: Severe myelosuppression with leukopenia and thrombocytopenia, which required transfusion of packed red blood cells and platelet concentrates.

Initial laboratory indicators at symptom onset: amylase 681 U/L, lipase 936.7 U/L, triglyceride 1.39 mmol/L, albumin 29 g/L, white blood cell count 0.66 × 10⁹/L, platelet count 27 × 10⁹/L, C-reactive protein (CRP) 19.24 mg/L, interleukin-6 (IL-6) 50.36 pg/mL. Within 24 h after symptom onset, biochemical indicators deteriorated rapidly: CRP peaked at 229.13 mg/L, and serum triglyceride level surged to 22.89 mmol/L. The rapid triglyceride elevation was considered secondary to severe systemic inflammatory response syndrome (SIRS) disrupting lipid metabolic pathways. Contrast-enhanced abdominal CT revealed diffuse pancreatic swelling and extensive peripancreatic fluid collection; chest CT identified bilateral pleural effusions and inflammatory pulmonary edema induced by SIRS.

#### Standard supportive treatment and complete TPE operation parameters

Maximal standardized critical care was continuously provided for 24 h without measurable clinical improvement, including fasting, nasogastric decompression, continuous octreotide infusion, balanced crystalloid-colloid fluid resuscitation and broad-spectrum intravenous antibiotics. Single-session rescue TPE was subsequently carried out in accordance with standardized pediatric apheresis protocols, with detailed technical parameters listed below:

1. Vascular access: A 6.5 French dual-lumen internal jugular central venous catheter placed under real-time ultrasound guidance.
2. Total exchanged plasma volume: 1200 mL, equivalent to 1.5 times the patient’s total plasma volume calculated via the Kaplan formula.
3. Replacement fluid: Leukocyte-reduced fresh frozen plasma (FFP) as the sole replacement medium.
4. Anticoagulation regimen: Unfractionated heparin 60 IU/kg loading bolus, followed by maintenance infusion at 15 IU/kg/h; target activated clotting time (ACT) maintained between 180 and 240 s.
5. Total procedural duration: 3.5 h of continuous circuit operation.
6. Blood flow rate: Adjusted between 30 and 40 mL/min based on the patient’s hemodynamic tolerance.
7. Intraprocedural monitoring: Continuous real-time vital sign monitoring; no hypotension, hemorrhage or transfusion hypersensitivity reactions occurred during the whole process.

Single-session TPE was selected due to rapid relief of organ dysfunction within 24 h after treatment, which eliminated the need for additional apheresis cycles.

#### Dynamic changes of laboratory biomarkers before and after TPE

#### Clinical recovery process

Severe abdominal pain and vomiting disappeared six hours after TPE implementation. Respiratory distress improved rapidly, and non-invasive ventilation was completely discontinued 24 h after the procedure. Pancreatic enzymes, hypertriglyceridemia and circulating inflammatory cytokines decreased significantly (Table 1). Serial chest radiography and abdominal CT showed gradual absorption of pleural effusions, pulmonary edema and peripancreatic fluid collections. All manifestations of multi-organ dysfunction were fully relieved within seven days. The patient gradually resumed normal oral feeding, continued scheduled maintenance chemotherapy, and maintained MRD-negative remission at the 3-month outpatient follow-up without recurrent pancreatitis. Long-term follow-up will be extended to 12 months to observe potential delayed pancreatic endocrine and exocrine dysfunction.

**Table 1:** Serial quantitative biomarker measurements at key clinical time points Footnote: All detection indicators were tested using the unified laboratory detection platform of Liaocheng People’s Hospital; reference ranges are consistent with the institutional standard laboratory values. TPE, therapeutic plasma exchange

Biomarker (Institutional Reference Range)	Symptom Onset	24 h Supportive Care Before TPE	6 h Post-TPE	72 h Post-TPE	7 Days Post-TPE
Amylase (30–110 U/L)	681	742	316	124	78
Lipase (13–60 U/L)	936.7	1025	421	109	52
Triglyceride (0.3–1.7 mmol/L)	1.39	22.89	8.12	2.05	1.42
CRP (0–10 mg/L)	19.24	229.13	110.67	36.21	7.35
IL-6 (< 7 pg/mL)	50.36	102.75	41.22	12.08	4.11
Creatinine (30–60 µmol/L)	48	96	72	53	45
Platelet Count (100–300 × 10⁹/L)	27	23	31	89	165

## Discussion

Asparaginase-related pancreatitis occurs in 1%–3% of children receiving ALL chemotherapy; concurrent MODS raises mortality above 15%, mainly caused by delayed diagnosis and uncontrolled cytokine-mediated inflammatory cascades [4, 5]. Clinicians should closely monitor four core warning signs within 72 h after pegaspargase administration: intractable abdominal pain, significant elevation of pancreatic enzymes, rapid triglyceride increase, and early respiratory or renal dysfunction. Contrast-enhanced abdominal CT is the gold-standard imaging examination to confirm SAP and evaluate local peripancreatic complications [14]. Pegaspargase directly damages pancreatic acinar cells and triggers massive release of systemic pro-inflammatory mediators, driving the progression from localized pancreatic injury to global organ failure. TPE theoretically blocks this pathological cascade by removing harmful circulating mediators, yet ASFA guidelines and meta-analytic evidence do not support generalized apheresis for all pancreatitis patients [10]. In this case, TPE was only administered after maximal standardized supportive care failed to reverse life-threatening MODS.

### Potential mechanisms of clinical improvement

After MODS onset, the patient developed severe hypertriglyceridemia with a peak value of 22.89 mmol/L. TPE rapidly reduced circulating triglyceride levels, IL-6 and CRP, alleviating systemic inflammatory burden. Given the high molecular weight and long plasma half-life of pegaspargase, effective extracorporeal clearance of this drug cannot be achieved 48 h after infusion. Therefore, the therapeutic benefit mainly stems from the removal of lipid toxicants and pro-inflammatory cytokines rather than elimination of the chemotherapeutic agent itself. Limitation: No serum pegaspargase concentration monitoring was performed during hospitalization, so this inference is supported only by pharmacokinetic theory without direct laboratory verification.

### Rational interpretation of TPE’s therapeutic value

The inherent limitations of single-case observational research prevent definitive causal correlation between TPE and full clinical recovery. The patient received uninterrupted multi-system supportive care throughout hospitalization, including fasting, somatostatin analog infusion, individualized fluid resuscitation, antibacterial treatment and non-invasive respiratory support. Rapid clinical stabilization reflects the combined therapeutic effect of comprehensive critical care and apheresis-mediated mediator clearance. According to pediatric clinical registry data, spontaneous recovery from severe pegaspargase-induced MODS without TPE is extremely rare; only temporal correlation between apheresis and symptom relief can be confirmed, rather than independent efficacy of TPE alone.

### Comparative analysis with published pediatric literature

Most pediatric patients with pegaspargase-related SAP receive only conservative supportive treatment without apheresis intervention. Among patients requiring TPE for pancreatitis combined with MODS, nearly all published clinical cohorts report 3 to 4 consecutive apheresis cycles to reverse decompensated organ function. Reports documenting complete MODS remission after a single TPE session remain scarce in existing medical literature. The strengths of this case report include standardized diagnosis based on the 2012 Atlanta Classification and PELOD-2 scoring system, complete transparent apheresis operation data, and longitudinal quantitative laboratory indicators, which are often omitted in previous case reports. This manuscript strictly complies with CARE reporting guidelines to improve clinical reproducibility for pediatric hematology and critical care clinicians.

### Study Limitations

1. This research is a retrospective single-case study, and clinical and biochemical observations derived from one individual cannot be generalized to all pediatric ALL patients with asparaginase-induced SAP.
2. No matched control cohort was set up to separate the independent therapeutic contribution of TPE from concurrent multi-organ supportive interventions.
3. Serum pegaspargase concentration detection was not implemented, so the inference of minimal drug clearance via TPE lacks direct laboratory evidence.
4. The 3-month follow-up period is insufficient to assess long-term pancreatic endocrine and exocrine sequelae as well as delayed chemotherapy-related toxicities.
5. No parallel inflammatory cytokine monitoring data from patients without TPE intervention were obtained to quantitatively distinguish the anti-inflammatory effects of apheresis and supportive care.
6. Large prospective multi-center pediatric clinical trials are required to establish evidence-based criteria for TPE indications, optimal intervention timing and reasonable treatment cycles.

## Conclusion

Pegaspargase-induced SAP combined with persistent MODS is a rare but fatal chemotherapy adverse reaction during pediatric ALL induction and maintenance treatment. Timely diagnosis guided by the 2012 Revised Atlanta Classification and PELOD-2 organ dysfunction scoring system, combined with early multi-system supportive care including individualized respiratory support, constitutes the core management strategy.

Single-session TPE can rapidly reverse refractory life-threatening MODS when maximal standardized conventional supportive care fails. Nevertheless, apheresis should only be reserved as a rescue intervention for strictly selected critically ill pediatric patients; universal prophylactic or routine TPE cannot be recommended based on current meta-analytic data and ASFA consensus guidelines. This standardized case report provides complete clinical, laboratory, imaging and apheresis procedural data to enrich the limited pediatric research on high-risk asparaginase-induced severe pancreatitis accompanied by multi-organ failure.

## Supplementary Information

Supplementary Material 1. Figure 1. Axial contrast-enhanced abdominal CT (November 16, 2025): Diffuse pancreatic enlargement and extensive peripancreatic inflammatory fat stranding, consistent with SAP diagnostic criteria of the 2012 Revised Atlanta Classification.

Supplementary Material 2. Figure 2. Axial chest CT (November 17, 2025): Bilateral moderate pleural effusions induced by systemic inflammatory response syndrome.

Supplementary Material 3. Figure 3. Axial abdominal CT (November 17, 2025): Pancreatic parenchymal swelling, massive peripancreatic fluid collection and intraperitoneal ascites.

Supplementary Material 4. Figure 4. Anteroposterior chest radiograph before TPE (November 18, 2025): Bilateral pleural effusions and diffuse inflammatory pulmonary edema; the patient received non-invasive ventilation at this time point.

Supplementary Material 5. Figure 5. Anteroposterior chest radiograph 60 hours after TPE (November 20, 2025): Complete absorption of right-sided pleural effusion and obvious relief of pulmonary edema; non-invasive ventilation was discontinued on this day.

Supplementary Material 6. Figure 6. Anteroposterior chest radiograph (November 23, 2025): Full absorption of bilateral pleural effusions and significant improvement of pulmonary inflammatory infiltration.

Supplementary Material 7. Figure 7. Axial contrast-enhanced abdominal CT (December 6, 2025): Mitigated pancreatic swelling with a tiny encapsulated fluid collection localized at the pancreatic tail; peripancreatic fluid accumulation was almost completely absorbed.

## Data Availability

No datasets were generated or analysed during the current study.

## Abbreviations

ALL: Acute Lymphoblastic Leukemia
SAP: Severe Acute Pancreatitis
MODS: Multiple Organ Dysfunction Syndrome
TPE: Therapeutic Plasma Exchange
CRP: C-reactive protein
IL-6: Interleukin-6
CT: Computed Tomography
PELOD-2: Pediatric Logistic Organ Dysfunction-2
FFP: Fresh Frozen Plasma
TPV: Total Plasma Volume
ACT: Activated Clotting Time
ASFA: American Society for Apheresis
MRD: Minimal Residual Disease
SIRS: Systemic Inflammatory Response Syndrome
PICU: Pediatric Intensive Care Unit
